# Gender differences in work–family conflict and mental health of Swedish workers by childcare responsibilities: findings from the SLOSH cohort study

**DOI:** 10.5271/sjweh.4231

**Published:** 2025-09-01

**Authors:** Yamna Taouk, Tania King, Constanze Leineweber, Brendan Churchill, Leah Ruppanner, Linda Magnusson Hanson

**Affiliations:** 1 Melbourne School of Population and Global Health, University of Melbourne, Melbourne, Australia.; 2 Division of Psychobiology and Epidemiology, Department of Psychology, Stockholm University, Stockholm, Sweden.; 3 School of Social and Political Sciences, University of Melbourne, Melbourne, Australia.

**Keywords:** childcaring intensity, depressive symptom, family-to-work conflict, work-to-family conflict

## Abstract

**Objectives:**

In Sweden, the number of working-aged women in employment is now almost equal to that of men. While this has many benefits, it presents challenges in organizing work and non-work responsibilities around children, which may impact employees' mental health.

**Methods:**

Based on the Swedish Longitudinal Occupational Survey of Health (SLOSH) cohort study, we prospectively examined gender differences for the effects of work–family conflict and caring for children on mental health among Swedish workers. Mental health status was assessed using a brief (Hopkins) symptom checklist depression scale. We used information from three waves of data over five years (2014–2018) for 5846 women and 4219 men aged 20–64 at baseline. Linear fixed-effects analyses were performed examining within-person changes in work interfering with family (WFC) and family interfering with work (FWC) and associated changes in depressive symptoms by childcare intensity (0, 1–10, >10 hours/week) and sex.

**Results:**

Changes in mean scores for WFC and FWC were associated with changes in depressive symptoms for men [no childcaring: WFC 1.31 (95% confidence interval (CI) 1.13–1.49), FWC 0.70 (95% CI 0.43–0.96); childcaring >10 hours/week: WFC 1.39 (95% CI 0.53–2.25), FWC 1.24 (95% CI 0.27–2.21)] and women [no childcaring: WFC 1.57 (95% CI 1.41–1.73), FWC 1.04 (95% CI 0.79–1.30); childcaring >10 hours/week: WFC 2.04 (95% CI 1.36–2.73), FWC 1.57 (95% CI 0.82–2.32)].

**Conclusion:**

Higher levels of WFC and FWC are associated with increased depressive symptoms in both men and women. The impact is greater for those with greater childcaring responsibilities intensity.

Over the past few decades, the landscape of work and family life has undergone significant changes, particularly in industrialized nations like Sweden. With rising female labor force participation and shifts in family structures, balancing work and family responsibilities has become increasingly complex for both men and women. In Sweden, labor force participation among women aged 15–64 has risen steadily from 59% in 1970 to 80% in 2020, bringing it nearly on par with men (1). Additionally, women’s employment is typically full-time or at the higher end of part-time work (ie, 26–35 hours/week) (2). While this development has brought numerous benefits, such as empowerment of women and improved family income, it has also intensified the pressures both women and men face in balancing work and non-work responsibilities. This challenge is often referred to broadly as ‘work–family conflict’, defined as a form of inter-role conflict where the demands of work and family roles are mutually incompatible, making it difficult for individuals to fulfil the requirements of both roles (3). Work–family conflict can negatively affect workers’ health and well-being. Evidence suggests that work–family conflict is associated with stress-related health outcomes such as insomnia, hypertension, alcohol abuse, and anxiety and depression (4–8).

Work–family conflict manifests in two primary directions: work interfering with family (WFC) and family interfering with work (FWC) (9). Both dimensions include time- and strain-based conflict. WFC may arise when work demands, such as long hours, spills into family life to limit the ability to fulfil family responsibilities or when the emotional and psychological stress from work negatively impacts on family life. Conversely, FWC occurs when family demands, such as leaving work early to pick up children from school, interfere with work responsibilities or when family issues, such as caring for children, affect work productivity. While WFC and FWC are reciprocally related, they can be viewed as distinct constructs (10). Though work-related factors are more strongly associated with WFC, and family-related factors are more closely linked to FWC (11), significant cross-domain correlations exist, and influences from one domain can impact the other (12). Both WFC and FWC have been found to negatively affect attitudes and behaviors, with individual role differences and situational factors moderating these effects (13).

With the rise in women’s employment, shifting societal expectations around gender roles, and both men and women experiencing increasing pressures of balancing work and family life, work–family conflict has become a more salient issue (14). However, the experience of work–family conflict is not uniform across all individuals; it is influenced by various factors, including gender, and caregiving responsibilities (15, 16). Research suggests that gender influences perceptions of work–family conflict, with the underlying assumption that WFC may place a greater burden on women than on men. This phenomenon is driven by traditional gender roles that often position men as primary breadwinners and women as primary caregivers. Although attitudes to gender roles have shifted, the unequal distribution of domestic and caregiving responsibilities continues to place a greater burden on women potentially leading to higher levels of FWC (17). Conversely, men may experience more work–family conflict due to societal expectations to prioritize work over family life (16). Furthermore, the effect of both WFC and FWC on health may differ by gender. The effect of WFC on mental health may be stronger among women than men (18). Women have historically prioritized household and childcare responsibilities. As a result, conflicts that impact family roles and cause family stress may have a more significant negative impact on women’s mental health compared to men. In contrast, FWC may impact men’s mental health. Traditionally, men have prioritized their role as breadwinners, so any conflict that interferes with their work responsibilities may have a more detrimental effect on their mental health compared to women (19).

The degree of childcaring responsibilities, such as the number of hours spent caring for young children, can further influence the extent of work–family conflict and its impact on mental health (6, 17). It signals the extent to which mothers and fathers have stepped into the role of primary carer, with longer hours engaged in caregiving signaling a more concrete primary caregiving role. Across countries and cultural contexts there are considerable variations in the availability of family-friendly work arrangements, such as flexible working hours or childcare services, as well as differences in endorsement of gender-related social norms including attitudes toward the division of household labor. Such variations may result in differences between countries in the levels of WFC and FWC and their impact on mental health.

In Sweden, the introduction of progressive family policies, such as generous parental leave, subsidized childcare and rearrangement of work time for parents of small children, aimed at supporting the combination of work and children, and actively promoting gender equality in both family life and the labor market have been implemented since the 1970s (2, 20). The high labor force participation rate of Swedish women, one of the highest by international standards, and the near parity in employment between working-aged women and men (1) is at least partly explained by such family-friendly working conditions. This milestone reflects a commitment to gender equality and has numerous positive implications for societal progress. However, the convergence of employment rates also brings forth challenges in balancing work and non-work responsibilities, particularly concerning childcare. Societal expectations for women to excel as both employees and parents, along with the pressure to balance these roles, often create an overwhelming burden that contributes to increased depressive symptoms (21). The increased participation of both parents in the workforce poses potential strains on organizing family life, potentially impacting the mental health of individuals, especially when managing the demands of employment alongside childcare responsibilities. As work and family dynamics evolve, addressing these challenges becomes crucial for sustaining the well-being of individuals and fostering a supportive environment where both genders can thrive in both their professional and personal roles. This necessitates a nuanced approach to work–life balance and family support policies that acknowledge the evolving landscape of gender roles in the workplace and at home.

This study aims to explore the gender differences in WFC and FWC and associations with mental health among Swedish workers, focusing specifically on the role of childcare responsibilities. By examining how WFC and FWC affect men and women differently, especially in the context of caregiving, this research seeks to provide insights into the underlying dynamics that contribute to mental health disparities in the workforce.

## Methods

**Figure 1:**
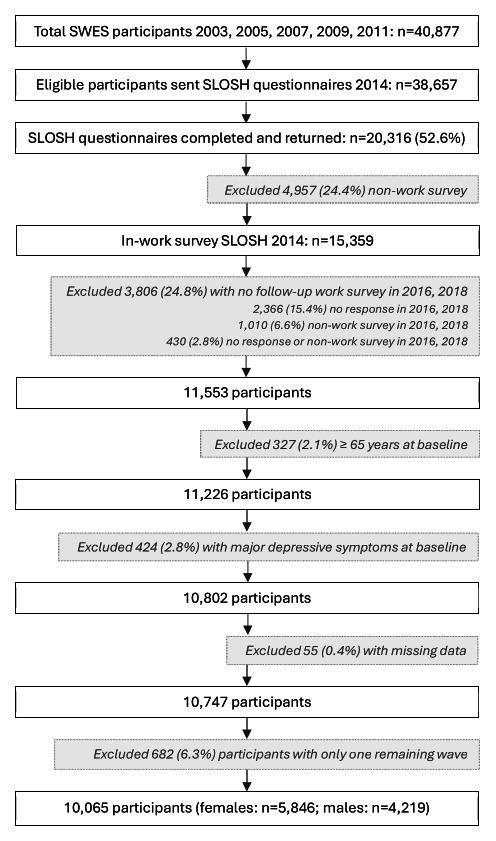
Flow chart illustrating the selection of the final analytic sample. SWES=Swedish Work Environment Survey; SLOSH=Swedish Longitudinal Occupational Survey of Health.]

Three waves of data from 2014 to 2018 of the biennial Swedish Longitudinal Occupational Survey of Health (SLOSH) survey were used. The SLOSH cohort began in 2006, providing a unique opportunity to study occupational health longitudinally using data from a nationally representative sample of the Swedish working population (22). The SLOSH sample is drawn from respondents from the Swedish Work Environment Survey (SWES), who in turn are randomly selected from the Labour Force Survey (LFS) conducted biennially by Statistics Sweden. The SWES is based on a nationally representative sample of the working population in Sweden. All eligible SWES participants from 2003–2011 were invited to respond to the SLOSH questionnaires 2014–2018. A detailed description of the selection procedure has been published elsewhere (22). Of the 40 877 SWES participants, 38 657 eligible individuals received SLOSH questionnaires in 2014, and 20 316 (52.6%) completed and returned them (22). Of these, 15 359 participants who had worked ≥30% of full-time hours during the preceding three months completed the ‘in-work’ survey, while 4957 participants responded to the non-work survey (figure 1).

This study sample was limited to those participants who completed the work survey of the SLOSH survey between 2016 and 2018 (N=11 553), were <65 years of age at baseline (N=11,226), had no major depressive symptoms at baseline (N=10 802), and did not have missing values for outcome, exposures, and covariates variables (N=10 747). Finally, 682 participants were excluded because the aforementioned selections resulted in only one wave of the in-work SLOSH survey remaining for them. Their exclusion was necessary, as calculating changes in work–family conflict and mental health would not have been feasible for them. The final analytic sample included 10 065 individuals and 25 904 observations (women: N=5846 and 15 080 observations; men: N=4219 and 10 824 observations) (figure 1). SWES participants in the SLOSH cohort consist of more women than men; they tend to be older, are often married, and are more likely to be Swedish. These characteristics also predict repeated participation in the SLOSH survey; those lost to follow-up are more often men, younger, unmarried, and/or not born in Sweden (22).

### Main exposures

*Work–family conflict.* The work–family conflict subscale in SLOSH is adapted from the work and non-work interference questionnaire by Fisher et al (23). Four items were used to estimate WFC, with the possible responses: (i) "When I get home from work, I’m too tired to do the things I’d like to do", (ii) "My private life doesn’t look like I would like it to because of my work", (iii) "I often overlook personal needs due to the demands of my work", and (iv) "My private life suffers because of my work". These were scored on a Likert scale ranging from 1 (not at all) to 5 (always), summed and an average score calculated with higher scores indicating greater WFC. The mean WFC score 2.62, standard deviation (SD) 0.97. For those participants missing data for one item (0.4%), scale scores were based on the average of the three completed items (24). Cronbach’s α for the four items relating to WFC domain was 0.90.

*Family–work conflict.* Three items were used to estimate FWC, with the possible responses: (i) "My work suffers because of everything going on in my personal life", (ii) "I would devote more time to work if it weren’t for everything I have going on in my personal life", and (iii) "I am too tired to be effective at work because of things I have going on in my personal life". These were scored on a Likert scale ranging from 1 (not at all) to 5 (always), summed and an average score calculated, with higher scores indicating greater FWC. The mean FWC score was 1.45, standard deviation (SD) 0.56. For those participants missing data for one item (0.3%), scale scores were based on the average of the two completed items (24). Cronbach’s α for the four items relating to FWC domain was 0.77.

### Outcome

Depressive symptoms were assessed using a brief version of the Hopkins Symptom Checklist (SCL) core depression scale (SCL-CD_6_) (25). The scale measures one-week prevalence and includes six items covering the core symptoms of depression: lowered mood, loss of interest, reduced energy, marked tiredness, excessive worries and self-accusation. This scale is particularly suitable for assessment in large population surveys because of its brevity and ease of administration and has been clinically validated (26). Items were scored on a five-point Likert scale ranging from 0 (not at all) to 4 (very much/extremely). Scores were summed and ranged from 0–24 with higher scores indicating worse mental health. A score of ≥17 has been calculated as the best cut-point for major depression (sensitivity 0.68, specificity 0.98), and significantly predicts subsequent use of antidepressants and hospitalizations for depressive episodes (27). Participants with scores of ≥17 at baseline were excluded from the analysis.

### Covariates

We controlled for possible prior common causes of work–family conflict and depressive symptoms. Relevant confounders included age (5-year bands) and survey year. The sociodemographic factors consisted of marital status, socio-economic status (SES) and total household income quintiles (1=lowest; 5=highest). Marital status was categorized as single or married/cohabiting. SES was based on the Swedish socio-economic classification (SEI), which incorporates occupational level and educational attainment (28), and was categorized as unskilled employees, skilled employees, assistant non-manual employees, intermediate non-manual employees, professionals and upper-level executives, self-employed and farmers. The presence of children ≤12 years in the household (yes/no) was controlled for in the analyses, as it could potentially confound the relationship between work–family conflict and mental health.

The number of hours per week spent on children’s activities – such as pick up, drop off, homework, care and supervision of children – and housework were collapsed into three categories (0, 1–10, >10 hours/week) to create childcare intensity and household variables. The number of hours worked in paid employment per week was classified into three categories (1–19, 20–40, and >40 hours/week). As housework and paid work hours could be construed as part of the work–family conflict exposures as well as potential confounders, additional analyses without adjustment for total working hours and housework hours per week were performed as sensitivity analyses. Health is a very strong predictor of depressive symptoms. General self-rated health, a widely used measure of perceived current health status, which has been shown to predict mortality (29), was assessed from a single-item five-point Likert scale question “How would you rate your general state of health” with responses ranging from 1=very good to 5=very bad. It is plausible that health is a confounder, mediator or a combination of both for work–family conflict and depressive symptoms. Hence, the inclusion of general health as an additional confounder was examined in additional analyses.

### Statistical analyses

Descriptive analyses were conducted to examine the sample characteristics. Longitudinal linear fixed-effects regression models were used to estimate within-person changes in depressive symptoms in relation to changes in mutually adjusted WFC and FWC. Utilizing fixed-effect models reduces potential bias by allowing for time-invariant confounding characteristics (such as unmeasured personality traits and attitudes associated with work–family conflict and mental health) to be effectively controlled for, with each person serving as their own control hence enhancing exchangeability between exposed and unexposed groups (30).

We investigated changes in WFC and FWC and associated changes in depressive symptoms separately for male and female workers as a comparison of the regression models with an interaction term between both WFC and sex, and FWC and sex, indicated an effect modification of the relationship between work–family conflict and mental health by sex (P=0.005 and P<0.001 respectively). We also tested effect modification of the relationship between work–family conflict and mental health by childcare intensity. Comparison of the regression models with an interaction term between time-varying work–family conflict variables and time-varying childcare intensity, using likelihood ratio tests, indicated strong effect modification of the relationship between WFC and depressive symptoms (P<0.001) and FWC and depressive symptoms (P<0.001) by childcare intensity for men, and some evidence of effect modification for female workers (P=0.01 and P=0.17 respectively). Hence all analyses were stratified by sex and level of childcaring intensity. Due to concerns about selective non-response or attrition based on mental health status, additional sensitivity analyses were conducted, including participants with major depressive symptoms at baseline in the analyses. All analyses were performed using Stata/SE version 18.0 (StataCorp, College Station, TX, USA).

## Results

The characteristics of the analytic sample are described in table 1. Almost all participants were born in Sweden, and most were married or registered as cohabiting with a partner. Around three-quarters of participants were aged 45–64 years, and 58% were women. Approximately half were represented in the upper two income quintiles, with three-fifths classified as intermediate non-manual employees or professionals/upper-level executives. Most participants reported very-to-fairly-good general health (83% women; 82% men) and working 20–40 hours per week (86% women; 81% men). Notably, 15% of men worked >40 hours per week. Most participants (76% of women; 73% of men) had no children ≤12 years in the household. Of women, 29% spent 1–10 hours per week caring for children and 9% spent >10 hours. A third of men spent 1–10 hours per week on childcaring responsibilities and 8% spent >10 hours. In total, 35% of women and 19% of men reported doing >10 hours of housework per week, and 66% of women and 44% of men in 'the >10 hours per week childcaring intensity' category spent >10 hours on housework.

**Table 1:** Sample characteristics at baseline.

Characteristics		Women N=5846 (58%)								Men N=4219 (42%)						
		Childcaring intensity (hours/week)						Total		Childcaring intensity (hours/week)						Total
		0 N=3631 (62.1%)		1–10 N=1675 (28.7%)		>10 N=540 (9.2%)				0 N=2515 (59.6%)		1–10 N=1372 (32.5%)		>10 N=332 (7.9%)		
		N (%)		N (%)		N (%)		N (%)		N (%)		N (%)		N (%)		N (%)
Age (years)																
	20–24	13 (0.4)		1 (0.1)		0 (0.0)		14 (0.2)		7 (0.3)		0 (0.0)		0 (0.0)		7 (0.2)
	25–29	105 (2.9)		10 (0.6)		8 (1.5)		123 (2.1)		67 (2.7)		6 (0.4)		9 (2.7)		82 (1.9)
	30–34	135 (3.7)		57 (3.4)		94 (17.4)		286 (4.9)		103 (4.1)		51 (3.7)		48 (14.5)		202 (4.8)
	35–39	100 (2.8)		205 (12.2)		155 (28.7)		460 (7.9)		109 (4.3)		140 (10.2)		90 (27.1)		339 (8.0)
	40–44	142 (3.9)		414 (24.7)		167 (30.9)		723 (12.4)		118 (4.7)		310 (22.6)		89 (26.8)		517 (12.3)
	45–49	397 (10.9)		533 (31.8)		89 (16.5)		1019 (17.4)		239 (9.5)		384 (28.0)		68 (20.5)		691 (16.4)
	50–54	838 (23.1)		279 (16.7)		23 (4.3)		1140 (19.5)		479 (19.1)		282 (20.6)		24 (7.2)		785 (18.6)
	55–59	1160 (31.9)		132 (7.9)		2 (0.4)		1294 (22.1)		767 (30.5)		146 (10.6)		4 (1.2)		917 (21.7)
	60–64	741 (20.4)		44 (2.6)		2 (0.4)		787 (13.5)		626 (24.9)		53 (3.9)		0 (0.0)		679 (16.1)
Country of birth																
	Sweden	3409 (93.9)		1572 (93.9)		510 (94.4)		5491 (93.9)		2369 (94.2)		1285 (93.7)		316 (95.2)		3970 (94.1)
	Nordic countries	129 (3.6)		36 (2.1)		7 (1.3)		172 (2.9)		60 (2.4)		31 (2.3)		2 (0.6)		93 (2.2)
	Rest of Europe	51 (1.4)		36 (2.1)		13 (2.4)		100 (1.7)		54 (2.1)		30 (2.2)		10 (3.0)		94 (2.2)
	Rest of the world	42 (1.2)		31 (1.9)		10 (1.9)		83 (1.4)		32 (1.3)		26 (1.9)		4 (1.2)		62 (1.5)
Married/cohabiting		2681 (73.8)		1414 (84.4)		485 (89.8)		4580 (78.3)		1913 (76.1)		1244 (90.7)		313 (94.3)		3470 (82.2)
Quintiles household income																
	1	838 (23.1)		174 (10.4)		48 (8.9)		1060 (18.1)		450 (17.9)		79 (5.8)		27 (8.1)		556 (13.2)
	2	668 (18.4)		211 (12.6)		76 (14.1)		955 (16.3)		539 (21.4)		191 (13.9)		62 (18.7)		792 (18.8)
	3	609 (16.8)		370 (22.1)		144 (26.7)		1123 (19.2)		457 (18.2)		335 (24.4)		86 (25.9)		878 (20.8)
	4	768 (21.2)		431 (25.7)		149 (27.6)		1348 (23.1)		520 (20.7)		348 (25.4)		81 (24.4)		949 (22.5)
	5	748 (20.6)		489 (29.2)		123 (22.8)		1360 (23.3)		549 (21.8)		419 (30.5)		76 (22.9)		1044 (24.7)
Socioeconomic status																
	Unskilled employees	439 (12.1)		151 (9.0)		25 (4.6)		615 (10.5)		494 (19.6)		149 (10.9)		32 (9.6)		675 (16.0)
	Skilled employees	551 (15.2)		186 (11.1)		24 (4.4)		761 (13.0)		512 (20.4)		230 (16.8)		37 (11.1)		779 (18.5)
	Assistant non-manual employees	630 (17.4)		239 (14.3)		67 (12.4)		936 (16.0)		253 (10.1)		105 (7.7)		38 (11.5)		396 (9.4)
	Intermediate non-manual employees	1322 (36.4)		686 (41.0)		251 (46.5)		2259 (38.6)		694 (27.6)		418 (30.5)		107 (32.2)		1219 (28.9)
	Professionals/Upper-level executives	673 (18.5)		410 (24.5)		168 (31.1)		1251 (21.4)		524 (20.8)		447 (32.6)		114 (34.3)		1085 (25.7)
	Self-employed/Farmers	16 (0.4)		3 (0.2)		5 (0.9)		24 (0.4)		38 (1.5)		23 (1.7)		4 (1.2)		65 (1.5)
Child ≤12 years in household		4 (0.1)		872 (52.1)		504 (93.3)		1380 (23.6)		20 (0.8)		825 (60.1)		303 (91.3)		1148 (27.2)
Paid work hours/week																
	<20	266 (7.3)		124 (7.4)		37 (6.8)		427 (7.3)		111 (4.4)		51 (3.7)		9 (2.7)		171 (4.1)
	20–40	3115 (85.8)		1406 (83.9)		481 (89.1)		5002 (85.6)		2026 (80.6)		1097 (80.0)		279 (84.0)		3402 (80.6)
	>40	250 (6.9)		145 (8.7)		22 (4.1)		417 (7.1)		378 (15.0)		224 (16.3)		44 (13.3)		646 (15.3)
Housework hours/week																
	0	8 (0.2)		0 (0.0)		0 (0.0)		8 (0.1)		33 (1.3)		1 (0.1)		0 (0.0)		34 (0.8)
	1–10	2613 (72.0)		1013 (60.5)		184 (34.1)		3810 (65.2)		2133 (84.8)		1073 (78.2)		185 (55.7)		3391 (80.4)
	>10	1010 (27.8)		662 (39.5)		356 (65.9)		2028 (34.7)		349 (13.9)		298 (21.7)		147 (44.3)		794 (18.8)
General health																
	Very good	1078 (29.7)		508 (30.3)		153 (28.3)		1739 (29.7)		609 (24.2)		374 (27.3)		93 (28.0)		1076 (25.5)
	Fairly good	1915 (52.7)		916 (54.7)		298 (55.2)		3129 (53.5)		1425 (56.7)		790 (57.6)		183 (55.1)		2398 (56.8)
	Neither good nor bad	443 (12.2)		187 (11.2)		68 (12.6)		698 (11.9)		371 (14.7)		169 (12.3)		46 (13.9)		586 (13.9)
	Pretty/very bad	195 (5.4)		64 (3.8)		21 (3.9)		280 (4.8)		110 (4.4)		39 (2.8)		10 (3.0)		159 (3.8)

Mean WFC scores were consistent across all childcaring intensity categories for women (mean 2.68, SD 0.98) and men (mean 2.55, SD 0.92) (table 2). Mean FWC scores increased across childcaring intensity categories for both women and men, however men had consistently higher FWC scores (table 2). Women reported higher depressive symptoms scores with increasing childcaring intensity, especially in >10 hours per week group (mean 5.92, SD 4.32), and men had lower depressive symptoms scores on average (mean 4.31, SD 4.00) (table 2).

**Table 2:** Distribution of work-to-family conflict (WFC), family-to-work conflict (FWC) and depressive symptoms by sex and childcaring intensity at baseline. [SD=standard deviation.]

Characteristics	Women N=5846 (58%)								Men N=4219 (42%)						
	Childcaring intensity (hours/week)						Total		Childcaring intensity (hours/week)						Total
	0		1–10		>10				0		1–10		>10		
	Mean (SD)		Mean (SD)		Mean (SD)		Mean (SD)		Mean (SD)		Mean (SD)		Mean (SD)		Mean (SD)
WFC	2.67 (0.99)		2.69 (0.98)		2.72 (0.92)		2.68 (0.98)		2.51 (0.93)		2.62 (0.91)		2.61 (0.90)		2.55 (0.92)
FWC	1.32 (0.47)		1.45 (0.55)		1.71 (0.72)		1.39 (0.53)		1.41 (0.52)		1.64 (0.60)		1.80 (0.65)		1.51 (0.57)
Depressive symptoms	4.93 (4.33)		5.18 (4.26)		5.92 (4.32)		5.09 (4.32)		4.14 (3.94)		4.56 (4.08)		4.58 (4.03)		4.31 (4.00)

Results of the fixed effects models, examining changes in work–family conflict and associated changes in depressive symptoms by childcaring intensity for women are presented in table 3. Model 1 included mutually adjusted WFC and FWC conflict variables, age, year of survey, marital status, household income, socioeconomic status, and child ≤12 years in household. On average, a one unit increase in WFC was associated with 1.57 (95% CI 1.41–1.73) unit increase in depressive symptoms among women with no childcaring responsibilities; 1.64 (95% CI 1.37–1.91) unit increase in depressive symptoms for women 1–10 childcare hours/week; and 2.04 (95% CI 1.36–2.73) unit increase in depressive symptoms for women >10 childcare hours/week. On average, a one unit increase in FWC was associated with 1.04 (95% CI 0.79–1.30) unit increase in depressive symptoms among women with no childcaring responsibilities; 1.27 (95% CI 0.84–1.70) unit increase in depressive symptoms for women 1–10 childcare hours/week; and 1.57 (95% CI 0.82–2.32) for women >10 childcare hours/week. Further controlling for general health in model 2 attenuated effect estimates only slightly, with results essentially unchanged.

**Table 3:** Fixed effects models examining changes in depressive symptoms and changes in work–family conflict (WFC) and family-to-work conflict (FWC) by childcare intensity for women [Coef.=coefficient; CI=confidence interval].

Depressive symptoms		Childcaring intensity 0 hours/week (N=9925 observations)				Childcaring intensity 1–10 hours/week (N=4062 observations)				Childcaring intensity >10 hours/week (N=1093 observations)		
		Model 1 ^a^		Model 2 ^b^		Model 1 ^a^		Model 2 ^b^		Model 1 ^a^		Model 2 ^b^
		Coef. (95% CI)		Coef. (95% CI)		Coef. (95% CI)		Coef. (95% CI)		Coef. (95% CI)		Coef. (95% CI)
WFC		1.57 (1.41–1.73)		1.35 (1.20–1.50)		1.64 (1.37–1.91)		1.29 (1.02–1.56)		2.04 (1.36–2.73)		1.48 (0.83–2.14)
FWC		1.04 (0.79–1.30)		0.90 (0.66–1.13)		1.27 (0.84–1.70)		1.16 (0.76–1.55)		1.57 (0.82–2.32)		1.38 (0.65–2.11)
Paid work hours/week												
	<20	0.20 (-0.11–0.52)		0.18 (-0.12–0.48)		0.22 (-0.46–0.90)		0.14 (-0.48–0.77)		-0.05 (-1.28–1.19)		0.18 (-0.91–1.28)
	20–40	0.00 (reference)		0.00 (reference)		0.00 (reference)		0.00 (reference)		0.00 (reference)		0.00 (reference)
	>40	-0.05 (-0.28–0.18)		-0.02 (-0.25–0.20)		-0.05 (-0.47–0.37)		0.06 (-0.34–0.46)		-1.40 (-2.45–-0.36)		-1.35 (-2.34–-0.35)
Housework hours/week												
	0	0.00 (reference)		0.00 (reference)		0.00 (reference)		0.00 (reference)		omitted		omitted
	1–10	-0.49 (-2.58–1.59)		-0.79 (-2.67–1.08)		1.07 (0.39–1.74)		0.61 (-0.01–1.23)		0.00 (reference)		0.00 (reference)
	>10	-0.41 (-2.51–1.68)		-0.68 (-2.56–1.20)		1.08 (0.29–1.88)		0.60 (-0.14–1.34)		0.49 (-0.47–1.45)		0.56 (-0.38–1.49)
Child ≤12 years		-3.34 (-6.54–-0.13)		-3.23 (-6.18–-0.28)		-0.18 (-0.71–0.36)		-0.13 (-0.62–0.36)		-0.01 (-2.44–2.43)		0.58 (-1.37–2.52)
General health												
	Very good			0.00 (reference)				0.00 (reference)				0.00 (reference)
	Fairly good			0.97 (0.73–1.21)				1.41 (0.96–1.85)				1.22 (0.36–2.08)
	Neither good nor bad			2.53 (2.12–2.94)				2.64 (1.88–3.40)				4.15 (2.54–5.76)
	Pretty bad			4.05 (3.39–4.71)				5.79 (4.72–6.86)				6.49 (3.80–9.18)
	Very bad			5.90 (3.69–8.10)				13.4 (9.83–16.9)				4.06 (-0.77–8.89)

^a^ Model 1 adjusted for age, year of survey, marital status, household income, socioeconomic status, child ≤12 years in household, hours of housework, paid work hours. ^b^ Model 2 further adjusted for general health.

Results for men are presented in table 4. Increases in WFC were associated with greater depressive symptoms across all childcaring intensity groups, with the strongest association observed among men with 1–10 childcare hours/week [1.65 (95% CI 1.35–1.96)]. Increases in FWC were associated with increases in depressive symptoms particularly in the highest childcaring intensity group [1.24 (95% CI 0.27–2.21)]. Results were essentially unchanged when models were further adjusted for general health.

**Table 4:** Fixed effects models examining changes in depressive symptoms and changes in work-to-family conflict (WFC) and family-to-work conflicts (FWC) by childcare intensity for men. [Coef.= coefficient; CI=confidence interval].

Depressive symptoms		Childcaring intensity 0 hours/week (N=6804 observations)				Childcaring intensity 1–10 hours/week (N=3342 observations)				Childcaring intensity >10 hours per week (N=678 observations)		
		Model 1 ^a^		Model 2 ^b^		Model 1 ^a^		Model 2 ^b^		Model 1 ^a^		Model 2 ^b^
		Coef. (95% CI)		Coef. (95% CI)		Coef. (95% CI)		Coef. (95% CI)		Coef. (95% CI)		Coef. (95% CI)
WFC		1.31 (1.13–1.49)		1.13 (0.96–1.30)		1.65 (1.35–1.96)		1.48 (1.18–1.78)		1.39 (0.53–2.25)		1.14 (0.38–1.90)
FWC		0.70 (0.43–0.96)		0.73 (0.48–0.98)		0.49 (0.10–0.88)		0.47 (0.10–0.85)		1.24 (0.27–2.21)		1.16 (0.32–1.99)
Paid work hours/week												
	<20	0.53 (0.09–0.97)		0.46 (0.04–0.89)		-0.29 (-1.09–0.51)		-0.39 (-1.19–0.41)		-2.99 (-5.66–-0.31)		-3.40 (-6.00–-0.80)
	20–40	0.00 (reference)		0.00 (reference)		0.00 (reference)		0.00 (reference)		0.00 (reference)		0.00 (reference)
	>40	0.24 (0.01–0.47)		0.25 (0.03–0.47)		0.35 (-0.06–0.76)		0.31 (-0.09–0.71)		-0.92 (-2.03–0.18)		-0.78 (-1.85–0.29)
Housework hours/week												
	0	0.00 (reference)		0.00 (reference)		0.00 (reference)		0.00 (reference)		omitted		omitted
	1–10	0.45 (-0.07–0.98)		0.42 (-0.08–0.91)		-1.98 (-4.66–0.69)		-1.58 (-3.66–0.50)		0.00 (reference)		0.00 (reference)
	>10	0.48 (-0.17–1.12)		0.44 (-0.17–1.05)		-1.71 (-4.45–1.02)		-1.30 (-3.45–0.85)		-0.27 (-1.15–0.61)		-0.14 (-0.97–0.69)
Child ≤12 years		1.56 (-0.46–3.57)		1.55 (-0.35–3.44)		0.30 (-0.20–0.80)		0.35 (-0.13–0.83)		0.29 (-1.12–1.70)		0.60 (-0.99–2.20)
General health												
	Very good			0.00 (reference)				0.00 (reference)				0.00 (reference)
	Fairly good			0.43 (0.17–0.69)				0.73 (0.27–1.19)				0.46 (-0.52–1.44)
	Neither good nor bad			1.60 (1.19–2.00)				2.20 (1.46–2.94)				2.70 (0.64–4.76)
	Pretty bad			3.11 (2.35–3.87)				3.45 (2.17–4.72)				5.93 (2.74–9.11)
	Very bad			7.61 (4.89–10.3)				7.00 (3.98–10.0)				12.9 (10.3–15.4)

^a^ Model 1 adjusted for age, year of survey, marital status, household income, socioeconomic status, child ≤12 years in household, hours of housework, paid work hours. ^b^ Model 2 further adjusted for general health.

Analyses were repeated without adjusting models for paid working hours and housework hours separately for women and men (supplementary material, www.sjweh.fi/article/4231, tables S1 & S2). Results substantiated main findings with little change in effect estimates. The study’s findings remained robust in additional sensitivity analyses, including participants with major depressive symptoms at baseline in the analyses, with results essentially unchanged (supplementary tables S3 & S4).

## Discussion

In our study, we found that work–family conflict is associated with depressive symptoms among both women and men. Mutually adjusted WFC and FWC appear to affect the mental health of female and male workers independently of each other. Both men and women seem to suffer increasing depressive symptoms with increasing WFC. As expected, increases in WFC led to greater increases in depressive symptoms among women. There was a clear stepwise relationship between childcaring intensity and depressive symptoms among female workers, with effect estimates for WFC increasing as childcaring intensity increased. The association between WFC and depressive symptoms among women was considerably stronger than for men among those women with childcaring responsibilities >10 hours/week.

While men had higher FWC mean scores than women, associations between changes in FWC and depressive symptoms were consistently stronger among women than men. This contrasts with Frone’s (18) observation that FWC has a stronger effect on men’s mental health, though this difference may be due to variations in the time period and population studied. There was a clear stepwise relationship between childcaring intensity and depressive symptoms among female workers, with effect estimates for FWC increasing as childcaring intensity increased. The impact of FWC on depressive symptoms among males, while not as strong as observed among females, was greatest in the highest childcaring intensity group (>10 hours/week). This suggests that men with the highest levels of childcaring responsibilities may have more permeable boundaries between work and family life compared to those with fewer or no childcaring duties, allowing family-related demands to interfere with work nearly as much as they do for women in similar roles.

Our results align with the findings of the limited studies to date that have explored the relationship between work–family conflict and mental health. Although previous studies addressed broader outcomes or focused on highly specific groups, it is likely that the findings from this study could still be meaningfully compared to other relevant research, even if measures of work–family conflict differ. In a recent study of mostly male workers in an international manufacturing and service company serving the gas and oil industry sector in Bahrain, work–family conflict was found to have a negative effect on psychological well-being (31). An Australian longitudinal analysis found that when fathers moved into high work–family conflict, from one wave to the next, their mental health deteriorated, and those who reported persistent high work–family conflict over two waves reported the highest psychological distress (6). A longitudinal study examined WFC and FWC and depressive complaints among predominantly male Dutch employees, and cross sectional associations were found for both with depressive complaints (32). Further cross-lagged structural equation modelling showed a reciprocal association between FWC and depressive complaints, but not for WFC and depressive complaints. Although the authors controlled for gender, they did not evaluate its moderating effect, though gender differences are observed in work–family conflict (33) and depressive disorders (34). A previous study using data from the 2006–2008 Swedish Longitudinal Occupational Survey of Health survey examined WFC and indicators of major depression, including the use of antidepressants (35). The study found that the effect estimates differed by sex. Sex-stratified analyses revealed an excess risk of subsequent major depression associated with high WFC among women, while the risk estimates for antidepressant use were stronger for men. Although this study used the same data source as the current study, the analytic sample was drawn from earlier survey waves (2006 and 2008), and their measure of WFC relied on a single item.

### Strengths and limitations

Key strengths of this study include the data source and methodology used to rigorously examine the relationship between work–family conflict and mental health. The utilization of SLOSH allowed for robust longitudinal analysis, characterizing and measuring changes in work–family conflict and mental health over time and controlling for confounders. The use of fixed effects regression allowed for each person to act as their own control in assessing the association between changes in work–family conflict and changes in depressive symptoms. This meant that time invariant confounders such as personality characteristics and traits were controlled for in the analysis, mitigating bias due to confounding. This study provided the opportunity to observe dose-dependent relationships and small associations between work–family conflict and mental health with a high degree of precision. Even though we excluded major depressive symptoms at baseline and included general health as an additional confounder (which may be a mediator on the pathway), we still observed consistent associations for work–family conflict and mental health by childcare intensity for Swedish female and male workers. Our measure of depressive symptoms, the SCL-CD6, while self-reported, has been clinically validated and assessed as a meaningful dimensional measure of depression severity (27).

Measurements of both dimensions of work–family conflict were limited to those items available in SLOSH which may not have adequately captured the full content domain of WFC and FWC conflict. Additionally, we could not control for the participants’ partners employment status which may have influenced measurement of participants’ work–family conflict exposures. Nonetheless, the results of this study are consistent with prior research that has used different measures of both types of work–family conflict that vary in length (11). However, we cannot rule out misclassification of the exposure given the self-report nature of our measures. The use of self-reported measures may have introduced common method bias, potentially leading to spurious or inflated associations (36). However, this is unlikely to be a significant concern, as within-person fixed effects analyses help mitigates common method bias by treating each person as their own case and control. Moreover, work–family conflict has been found to be associated with both self-report and objective measures of health (37). Furthermore, measured time-invariant characteristics such as sex as well as unmeasured stable factors like personality and temperament, which may increase common method bias, were stratified or controlled for in our within-person analyses. It may be possible that that unmeasured confounders may have biased results of our study. The within-person fixed-effects analytic approach somewhat mitigates confounding bias by controlling for time-invariant confounding and through the inclusion of key time-varying confounders as covariates in the models, however we cannot rule out confounding due to unmeasured time-varying confounders. A potential limitation is that healthy-worker selection at baseline, along with accumulated health selection and attrition over time, may have led to an underestimation of the results. We cannot be certain that selective non-response or dropout based on mental health outcome did not influence the results. However, additional sensitivity analyses, including participants with major depressive symptoms at baseline in the analyses, suggest that this is not a major concern in this study. Generalisability of our results is limited to Swedish workers, and results may not be extended beyond the Swedish context and neighbouring Scandinavian countries with similar social and economic policies.

### Concluding remarks

In conclusion, work–family conflict is deleterious to the mental health of both male and female employees, and childcaring responsibility intensifies the impact. It appears that both WFC and FWC may affect mental health independently of each other. This suggests that work and family roles and the balance between the two are important for the mental health of male and female employees. This has policy implications. More attention needs to be devoted to identifying and implementing strategies to enable employees to cope with or reduce exposure to both types of work–family conflict. Employers should be concerned with FWC as a source of stress in the lives of their employees and as a potential liability in terms of health care costs and productivity. It is likely that many family-supportive programs or coping strategies have a primary impact on only one of the two forms of work–family conflict.

## Supplementary material

Supplementary material
