# Supplementary material for: Gender differences in work–family conflict and mental health of Swedish workers by childcare responsibilities: findings from the SLOSH cohort study
Source: Scand J Work Environ Health. 2025 Aug 29;51(5):413–22. doi: 10.5271/sjweh.4231 (PMC12414518; doi:10.5271/sjweh.4231)
Supplement: Supplementary material [file SJWEH-51-413-S001.pdf]

Gender differences in work-family conflict and mental health of Swedish workers by childcare responsibilities: findings from the SLOSH cohort study[1]

by Yamna Taouk, PhD,[2] Tania King, PhD, Constanze Leineweber, PhD, Brendan Churchill, PhD, Leah Ruppanner, PhD, Linda Magnusson Hanson, PhD

1. Supplementary material
2. Correspondence to: Dr Yamna Taouk, Melbourne School of Population and Global Health, University of Melbourne, Melbourne, Australia 3010. [E-mail: taouk.y@unimelb.edu.au]

Supplementary Table S1: Fixed effects models examining changes in depressive symptoms and changes in work-family conflict by childcare intensity for women (Coef.= coefficient; CI=confidence interval)

Depressive symptoms	Childcaring intensity 0 hours per week (n=9,925 observations)		Childcaring intensity 1-10 hours per week (n=4,062 observations)		Childcaring intensity >10 hours per week (n=1,093 observations)	
	Model 1 Coef. (95% CI)	Model 2 Coef. (95% CI)	Model 1 Coef. (95% CI)	Model 2 Coef. (95% CI)	Model 1 Coef. (95% CI)	Model 2 Coef. (95% CI)
WFC	1.56 (1.41 - 1.72)	1.35 (1.20 - 1.50)	1.63 (1.36 - 1.90)	1.29 (1.02 - 1.56)	1.92 (1.21 - 2.62)	1.35 (0.67 - 2.03)
FWC	1.05 (0.80 - 1.30)	0.90 (0.66 - 1.14)	1.28 (0.86 - 1.71)	1.16 (0.76 - 1.55)	1.57 (0.81 - 2.34)	1.41 (0.67 - 2.14)
Child ≤ 12 years	-3.33 (-6.53 - -0.13)	-3.22 (-6.17 - -0.28)	-0.18 (-0.71 - 0.36)	-0.13 (-0.62 - 0.36)	-0.23 (-2.57 - 2.11)	0.39 (-1.49 - 2.27)
General Health						
very good		0.00 (reference)		0.00 (reference)		0.00 (reference)
fairly good		0.97 (0.73 - 1.21)		1.41 (0.96 - 1.85)		1.26 (0.40 - 2.12)
neither good nor bad		2.53 (2.12 - 2.94)		2.64 (1.88 - 3.40)		4.05 (2.41 - 5.69)
pretty bad		4.05 (3.39 - 4.71)		5.79 (4.73 - 6.86)		6.70 (4.01 - 9.38)
very bad		5.90 (3.70 - 8.10)		13.4 (9.82 - 16.9)		5.32 (0.96 - 9.69)

Model 1 adjusted for age, year of survey, marital status, household income, socioeconomic status, and child ≤12 years in household

Model 2 further adjusted for general health

Supplementary Table S2: Fixed effects models examining changes in depressive symptoms and changes in work family conflict by childcare intensity for men (Coef.= coefficient; CI=confidence interval)

Depressive symptoms	Childcaring intensity 0 hours per week (n=6,804 observations)		Childcaring intensity 1-10 hours per week (n=3,342 observations)		Childcaring intensity >10 hours per week (n=678 observations)	
	Model 1 Coef. (95% CI)	Model 2 Coef. (95% CI)	Model 1 Coef. (95% CI)	Model 2 Coef. (95% CI)	Model 1 Coef. (95% CI)	Model 2 Coef. (95% CI)
WFC	1.32 (1.14 - 1.50)	1.14 (0.97 - 1.31)	1.69 (1.38 - 1.99)	1.51 (1.21 - 1.81)	1.37 (0.50 - 2.24)	1.12 (0.36 - 1.87)
FWC	0.70 (0.43 - 0.96)	0.73 (0.48 - 0.98)	0.50 (0.10 - 0.89)	0.48 (0.10 - 0.86)	1.38 (0.44 - 2.32)	1.32 (0.51 - 2.13)
Child ≤ 12 years	1.61 (-0.42 - 3.65)	1.61 (-0.31 - 3.52)	0.31 (-0.19 - 0.80)	0.36 (-0.12 - 0.84)	0.51 (-0.91 - 1.93)	0.82 (-0.82 - 2.47)
General Health						
very good		0.00 (reference)		0.00 (reference)		0.00 (reference)
fairly good		0.43 (0.17 - 0.69)		0.72 (0.26 - 1.17)		0.35 (-0.63 - 1.33)
neither good nor bad		1.60 (1.19 - 2.01)		2.19 (1.45 - 2.94)		2.79 (0.70 - 4.88)
pretty bad		3.12 (2.36 - 3.89)		3.48 (2.17 - 4.79)		5.65 (2.32 - 8.98)
very bad		7.62 (4.90 - 10.3)		7.27 (4.62 - 9.91)		12.4 (9.74 - 15.1)

Model 1 adjusted for age, year of survey, marital status, household income, socioeconomic status, and child ≤12 years in household

Model 2 further adjusted for general health

Supplementary Table S3: Fixed effects models examining changes in depressive symptoms and changes in work-family conflict by childcare intensity for women including women with clinical depression/anxiety symptoms at baseline (Coef.= coefficient; CI=confidence interval)

Depressive symptoms	Childcaring intensity 0 hours per week (n=10,391 observations)		Childcaring intensity 1-10 hours per week (n=4,250 observations)		Childcaring intensity >10 hours per week (n=1,169 observations)	
	Model 1 Coef. (95% CI)	Model 2 Coef. (95% CI)	Model 1 Coef. (95% CI)	Model 2 Coef. (95% CI)	Model 1 Coef. (95% CI)	Model 2 Coef. (95% CI)
WFC	1.75 (1.58 - 1.91)	1.49 (1.33 - 1.65)	1.76 (1.47 - 2.04)	1.38 (1.10 - 1.66)	2.07 (1.41 - 2.73)	1.64 (1.02 - 2.26)
FWC	1.10 (0.84 - 1.36)	0.94 (0.70 - 1.18)	1.34 (0.92 - 1.77)	1.22 (0.83 - 1.61)	1.31 (0.60 - 2.02)	1.38 (0.65 - 2.11)
Paid work hours/week						
<20	0.12 (-0.22 - 0.46)	0.10 (-0.22 - 0.43)	0.19 (-0.50 - 0.88)	0.08 (-0.55 - 0.71)	-0.01 (-1.21 - 1.20)	0.17 (-0.93 - 1.27)
20-40	0.00 (reference)	0.00 (reference)	0.00 (reference)	0.00 (reference)	0.00 (reference)	0.00 (reference)
>40	-0.13 (-0.37 - 0.11)	-0.09 (-0.32 - 0.15)	-0.02 (-0.46 - 0.41)	0.08 (-0.33 - 0.48)	-1.23 (-2.28 - -0.19)	-1.36 (-2.34 - -0.38)
Housework hours/week						
0 hours	0.00 (reference)	0.00 (reference)	0.00 (reference)	0 (reference)	omitted	omitted
1-10 hours	-0.33 (-2.59 - 1.93)	-0.67 (-2.70 - 1.36)	0.97 (0.29 - 1.65)	0.51 (-0.12 - 1.14)	0.00 (reference)	0.00 (reference)
>10 hours	-0.28 (-2.55 - 2.00)	-0.57 (-2.61 - 1.47)	1.07 (0.27 - 1.88)	0.59 (-0.15 - 1.34)	0.68 (-0.26 - 1.63)	0.74 (-0.16 - 1.65)
Child ≤ 12 years	-3.33 (-6.62 - -0.05)	-3.22 (-6.19 - -0.24)	-0.10 (-0.64 - 0.44)	-0.03 (-0.53 - 0.47)	0.31 (-2.07 - 2.69)	1.03 (-1.02 - 3.09)
General Health						
very good		0.00 (reference)		0.00 (reference)		0.00 (reference)
fairly good		1.04 (0.79 - 1.29)		1.41 (0.96 - 1.86)		1.25 (0.41 - 2.08)
neither good nor bad		2.64 (2.22 - 3.07)		2.96 (2.20 - 3.73)		3.93 (2.32 - 5.54)
pretty bad		4.56 (3.91 - 5.20)		5.78 (4.74 - 6.82)		5.06 (2.73 - 7.39)
very bad		6.68 (4.67 - 8.68)		11.6 (7.79 - 15.3)		5.77 (1.51 - 10.0)

Model 1 adjusted for age, year of survey, marital status, household income, socioeconomic status, child ≤12 years in household, hours of housework, paid work hours

Model 2 further adjusted for general health

Supplementary Table S4: Fixed effects models examining changes in depressive symptoms and changes in work family conflict by childcare intensity for men including men with clinical depression/anxiety symptoms at baseline (Coef.= coefficient; CI=confidence interval)

Depressive symptoms	Childcaring intensity 0 hours per week (n=6,967 observations)		Childcaring intensity 1-10 hours per week (n=3,427 observations)		Childcaring intensity >10 hours per week (n=701 observations)	
	Model 1 Coef. (95% CI)	Model 2 Coef. (95% CI)	Model 1 Coef. (95% CI)	Model 2 Coef. (95% CI)	Model 1 Coef. (95% CI)	Model 2 Coef. (95% CI)
WFC	1.36 (1.18 - 1.54)	1.18 (1.01 - 1.35)	1.68 (1.38 - 2.00)	1.49 (1.19 - 1.79)	1.40 (0.54 - 2.26)	1.17 (0.39 - 1.95)
FWC	0.71 (0.44 - 0.97)	0.73 (0.48 - 0.98)	0.54 (0.15 - 0.93)	0.50 (0.12 - 0.87)	1.16 (0.24 - 2.08)	1.16 (0.36 - 1.97)
Paid work hours/week						
<20	0.51 (0.07 - 0.96)	0.47 (0.04 - 0.90)	-0.35 (-1.16 - 0.45)	-0.48 (-1.29 - 0.33)	-2.83 (-5.12 - -0.54)	-2.71 (-5.17 - -0.25)
20-40	0.00 (reference)	0.00 (reference)	0.00 (reference)	0.00 (reference)	0.00 (reference)	0.00 (reference)
>40	0.21 (-0.02 - 0.44)	0.22 (-0.00 - 0.45)	0.31 (-0.10 - 0.72)	0.29 (-0.11 - 0.69)	-0.77 (-1.88 - 0.34)	-0.69 (-1.76 - 0.38)
Housework hours/week						
0 hours	0.00 (reference)	0.00 (reference)	0.00 (reference)	0.00 (reference)	0.00 (reference)	0.00 (reference)
1-10 hours	0.40 (-0.13 - 0.92)	0.36 (-0.13 - 0.86)	-2.85 (-5.66 - -0.04)	-2.28 (-4.49 - -0.07)	-0.19 (-1.09 - 0.71)	-0.15 (-0.99 - 0.70)
>10 hours	0.46 (-0.19 - 1.10)	0.41 (-0.20 - 1.03)	-2.50 (-5.36 - 0.37)	-1.95 (-4.23 - 0.33)	omitted	omitted
Child ≤ 12 years	1.53 (-0.47 - 3.53)	1.52 (-0.35 - 3.40)	0.18 (-0.33 - 0.69)	0.25 (-0.24 - 0.74)	0.33 (-1.08 -, 1.74)	0.57 (-0.94 - 2.08)
General Health						
very good		0.00 (reference)		0.00 (reference)		0.00 (reference)
fairly good		0.43 (0.17 - 0.70)		0.81 (0.35 - 1.28)		0.43 (-0.55 - 1.41)
neither good nor bad		1.70 (1.28 - 2.11)		2.37 (1.61 - 3.13)		2.13 (0.07 - 4.19)
pretty bad		3.28 (2.51 - 4.06)		3.88 (2.66 - 5.09)		6.15 (2.84 - 9.46)
very bad		6.60 (4.04 - 9.16)		5.97 (2.57 - 9.38)		12.1 (9.50 - 14.6)

Model 1 adjusted for age, year of survey, marital status, household income, socioeconomic status, child ≤12 years in household, hours of housework, paid work hours

Model 2 further adjusted for general health
